# Pesticide residues in *Rita rita* and *Cyprinus carpio* from river Ganga, India, and assessment of human health risk

**DOI:** 10.1016/j.toxrep.2021.08.013

**Published:** 2021-09-01

**Authors:** Zeshan Umar Shah, Saltanat Parveen

**Affiliations:** Limnology Research Laboratory, Department of Zoology, Aligarh Muslim University, Aligarh, 202002, India

**Keywords:** Health risk assessment, *Rita rita*, *Cyprinus carpio*, Pesticides, Accumulation, River Ganga

## Abstract

Present study was carried out to determine the concentration and bioaccumulation of pesticide residues in two commonly edible fishes: bagrid fish, *Rita rita* and common carp, *Cyprinus carpio* collected from river Ganga at Narora, India. The human health risk via consumption of these fishes was also assessed. The n-hexane extract of the muscle tissues was characterized by gas chromatography coupled to mass spectrometry and quantified by electron capture detector for pesticide residues. Bioaccumulation factor (BAF) in bagrid fish for detected pesticides was found to be higher than those in common carp. Estimated daily intake (EDI) values in our study were insignificantly higher than Average daily intake (ADI) values. Target hazard quotient (THQ) via consumption of selected fishes was found to be lower than the set 1.0, inferring non-carcinogenic risk. With regard to contaminants carcinogenic effects the total risk ratio (R) values of each pesticide was found lower than threshold risk limit except of heptachlor which indicates carcinogenic risk. The results justify pesticide pollution in river Ganga at Narora and thus more attention is required in order to help improve the health status of this ecosystem and reduce contamination of fishes.

## Highlights

• Accumulation of pesticide residues in fish due to continuous use in agricultural areas along river Ganga basin.
• Residues concentrate in the humans through consumption of food from the river.
• Health impacts like non-carcinogenic (THQ) and carcinogenic (R) risks are never negligible by these residues.

## Graphical abstract

**Figure:**
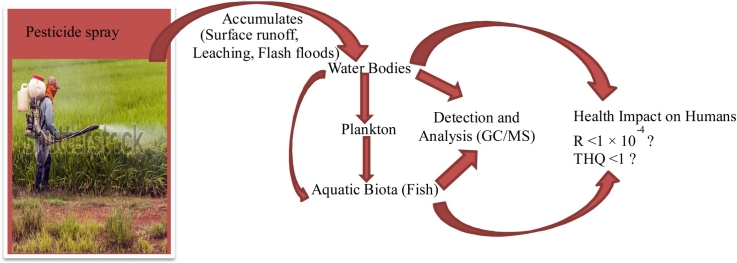

## 1 Introduction

Pesticides are widely used in the world to control various pests in the crops. After application the pesticide residues tend to persist and enter the aquatic ecosystem organisms where they accumulate. Various studies have correlated the pesticide exposure with allergies, cancer, neuro abnormalities, endocrine dysfunctioning, abnormal physiology, developmental effects, headaches, stomachaches, vomiting, skin rash, coma etc., [[1], [2], [3], [4], [5], [6]]. Regular consumption of food from pesticide contaminated source has both in-short duration (acute) and long-duration (chronic) effects. Acute pesticide poisoning has now become a rare evident however, long chronic toxicity caused by long duration exposure to low dose are commonly evident [6].

The river Ganga, the largest source of drinking water and irrigation in India also provides basic nutrition to the population living along the areas [7]. With the increase in population India has been undergoing rapid industrialization and economic development. Use of pesticides in agricultural sector has increased to hundred times to sustain more population in the country. Enormous quantities of pesticides are being applied along the Ganga river basin in agricultural fields [[8], [9], [10]]. Their residues finally find their way into the river by flash floods, leaching, drainage and surface runoff. A large number of reports are available that show river Ganga is highly polluted [[11], [12], [13], [14], [15], [16]]. At the international level, many reports are also available that show that the pesticide residues are present in water resources [[17], [18], [19], [20]]. Presence of pesticide residues in the fish tissues [[21], [22], [23], [24], [25]] shows that these residues bio-accumulated along trophic level in the food chain.

In India, 60,000 MT of pesticides are being annually used of which maximum consumption occurs along river Ganga basin [26]. Besides the regular agricultural activities done along the Ganga basin, the dry beds of the river are used to grow vegetables and fruits, also add pesticides to the river during monsoon season.

Among all aquatic organisms fish is considered as suitable bio-indicator animal in monitoring environmental contamination. Fish uptakes pollutants directly through the water via gills, integuments, from the food intake and shows increased ability to bio-accumulate due to their lower mono-oxygenase (detoxifying enzyme) activity [27]. The pollutants present in the fish not only indicate persistence in the environment but also their transfer to other organisms through the food web. Fish as nutrition is an important source of not only proteins but also omega-3-polyunsaturated fatty acids which are recommended in cardiovascular diseases [28]. Besides fish fatty acids are used in the pharmaceutical and cosmetic preparations [29]. However, consumption of fish from the contaminated environment may causes accumulation of pollutants in the human body.

Pesticides risk assessment is given as a function of toxicological effects, that is usually expressed as the ratio of predicted environmental concentration and average daily consumption to average body weight. Around the globe various studies have been conducted to evaluate the health risk associated with consumption of pesticide contaminated fish [[30], [31], [32], [33], [34], [35], [36]] including number of studies from the country [[37], [38], [39]].

Therefore, for the present study we have chosen river Ganga at Narora as sampling station. The objective of the study was to estimate the concentration of pesticide residues in water and accumulation in two food fish species, bagrid catfish and common carp. Further the data were used to assess daily exposure and human health risk by the consumption of these fishes.

## 2 Materials and methods

### 2.1 Study site and the sampling

**Fig. 1:**
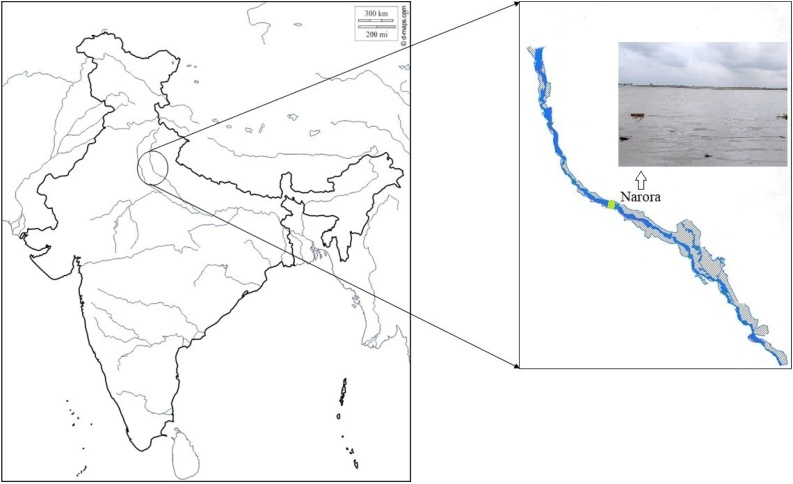
Sketch map of the sampling site.

Fig. 1 displays the details of sampling site, ten fish samples of each species and ten water samples were collected from Narora site (28º10ʹ59ʺN 78º23ʹ34ʺE) Uttar Pradesh of the river Ganga in August 2019. After collection the fishes were decapitated. Both the water and the fish samples were packed in ice-box and immediately brought to laboratory. The biometric data of both the fishes are given in Table 1.

**Table 1:** Biometric data of the selected fishes collected from River Ganga.

	Name of fish	
Parameters	Bagrid fish (*Rita rita*) (n = 10)	Common carp (*Cyprinus carpio*) (n = 10)
Fish length (cm)	22.13 ± 4.13	27.33 ± 6.42
Fish weight (g)	350.71 ± 20	380.66 ± 17
Dietary habit^a^	Carnivorous	Omnivorous
Trophic level^a^	3.7	3.1
Lipid % (Muscle)	14.36 ± 1.19	8.49 ± 3.68

a www. fish base org.

#### 2.1.1 Identification of fish

Fishes were identified following the keys given by Jhingran [40] and Fish Base org: -

*Rita rita*

Order: Siluriformes

Family: Bagridae

Fin formula: D 1 6; A ii 10-11; P 1 10; V I 6-7; Barbells 3 pairs

*Cyprinus carpio*

Order: Cypriniformes

Family: Cyprinidae

Fin formula: D 3-4/ 18-20; A 3-5; P_1_ 1/15; P_2_ 1/8

#### 2.1.2 Sample preparation

In the laboratory, fishes were dissected and dorsal muscle tissues of the fish were taken out for pesticide residue analysis. Collected muscle samples 10 g of each sample was freeze-dried, grounded to fine powder, and stored at −20 °C before the process of extraction. The impurity particulates in the collected water samples were separated by filtration through 0.45-μm hydrophilic filters.

### 2.2 Sample extraction and clean up

#### 2.2.1 Pesticides in water samples

Pesticides in water samples were analysed following the method of Muir and Sverko [41]. 2,4,5,6-tetrachloro-*m*-xylene (TCmX) as recovery surrogate was added in 1 L of filtered water sample. Liquid-liquid extraction with dichloromethane (35 mL) was performed. Na_2_SO_4_ column was used to remove water in the organic phase further n-hexane was used as an organic solvent. The column was packed from bottom to top with neutral silica, neutral alumina, and anhydrous sodium sulfate, to remove impurities in the extract. The extract was finally blown to dryness by purity nitrogen and the residues were redissolved with 20 μl of *n*-hexane.

#### 2.2.2 Pesticides in fish samples

**Fig. 2:**
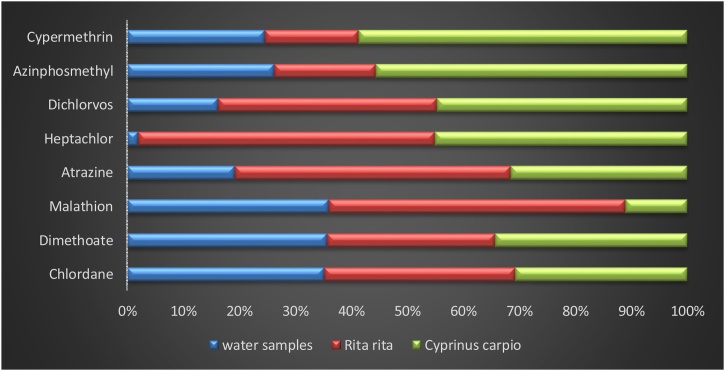
Relative abundance of pesticides in water, muscle tissues of *Rita rita* and *Cyprinus carpio*.

Soxhlet extraction method [42] was followed to extract pesticides in fish muscle. 10 g of muscle tissue after freeze dried were homogenized to fine powder with 35−40 g of activated anhydrous sodium sulfate. The prepared sample was packed and placed in extracting thimble of the Soxhlet apparatus. The mixture was extracted with 150 mL of acetone and n-hexane (20:80) v/v for 6 h. The extract was filtered and concentrated to 2 mL on water bath and transferred into (0.22 μm) membrane filter polyethylene (10 mL) syringe. The column was packed with neutral silica, acidic silica, florisil acidic alumina and sample mixture from bottom to top with other membrane filter placed on top. 15 mL of n-hexane was used to elute the packed column at a flow rate of 2 mL min^−1^. High purity nitrogen at the gentle stream was used to dry eluent, followed by dry residues redissolution with 200 μL of n-hexane and addition of internal standard (pentachloronitrobenzene). Qualitative as well as quantitative analysis of analytes were done with GC–MS and GC-ECD. Fig. 2 depicts the relative abundance of pesticides detected in water samples and muscle tissues of the two fishes.

### 2.3 Determination of lipid content

Determination of lipid content was done gravimetrically [43]. 2 g of fine grounded muscle powder was dissolved in twenty millilitres of water, cyclohexane and 16 mL isopropyl (3:1) mixture. Ultrasonic extraction was done, mixture reached statically separated equilibrium, and the organic phase was collected. Extraction was repeated with 18 mL of cyclohexane and 6 mL isopropyl alcohol and combined with earlier then dried under a gentle nitrogen stream. The residue was weighed and recorded in mg and the percentage content was calculated per gram tissue sample.

### 2.4 Qualitative and quantitative analysis of pesticide residues by GC–MS

3 μL of sample was injected into a gas chromatography (Agilent 7890A, USA) instrument equipped with an electron capture detector (GC-ECD) (Agilent Technologies, USA) the analytical capillary column was DB-5 (30 m ×0.25 mm i.d × 0.25-μm film thickness, Agilent, USA). Nitrogen was used as carrier gas with the flow rate of (1 mL min^−1^). Injector and detector temperatures were adjusted at 250 and 300 °C. Started at 80 °C with 1 min hold, and the oven temperature was raised to 150 °C at 20 °C min^−1^ rate and finally to 300 °C (5 min hold) at the rate of 5 °C min^−1^.

The instruments were calibrated with calibration standards during analysis. Each sample was analyzed in duplicate. The recoveries of TCmX (surrogate standard) were 75 ± 6 % in water samples and 68 ± 6 % in fish samples. The recoveries of pesticides ranged from 73 to 100 % in water samples and from 66 to 84 % in fish samples. The method detection limits (MDLs) concentration of analytes were confirmed whose signal-to-noise (S/N) ratio was three and ranged from 0.05–100 μg L^−1^ in water samples and from 0.01−100 μg g^−1^ in fish samples. Concentration detected less than MDLs in samples was treated as not detected (nd).

### 2.5 Data analysis

The values of pesticides in both fishes were statistically analysed by Spearman correlation test by using SPSS statistical package (version 16.0; SPSS Inc., USA).

Bio-water accumulation factor (BAF) illustrates the partitioning of chemical between water and aquatic organisms, it provides the accumulation-scale of the contaminants in the organism.

BAF is calculated by the following equation:

BAF=Cl/c

Where *C_l_* is the pollutant concentration in the fish (μg g^−1^) normalized by lipid content of fish and *c* is concentration of pollutant in water (μg l^−1^).

In order to determine potential human health risk of tested fishes, the estimate daily intake (EDI), target hazard quotients (THQ) and Carcinogenic risk (R) were calculated.

The target hazard quotients (THQs), and carcinogenic risk ratio (R) were used in risk assessment. The THQ > 1 denotes that the daily exposure may cause human health hazard effects.

The calculations of EDI [44], THQ, and R are done using the formulae [45]:

$$\text{E}\text{D}\text{I}=\frac{C\;\times \;W_{F}}{W_{AB}}$$

Where,

*C* = Concentration of pollutant in food (μg g^−1^), W_F_ = Average daily fish consumption in India is 55 g day^−1^ person^−1^, and *W*_AB_ = Average adult body weight (70 kg) ([45], Jiang et al. [46],[1]).

$$\text{T}\text{H}\text{Q}=\frac{E_{F}{\times E}_{D}{\times F}_{IR}\times C}{R_{FD}{\times W}_{AB}{\times T}_{A}}\;\times 10^{-3}$$

$$\text{R}=\frac{E_{F}{\times E}_{D}{\times F}_{IR}{\times S}_{F}\times C}{W_{AB}{\times T}_{A}}\;\times \;10^{-3}$$

Where,

*E*_F_ = frequency of exposure (350 days year^−1^), *E*_D_ = duration of exposure (70 years), *F*_IR_ = Average daily fish consumption in India is 55 g day^−1^ person^−1^, *R*_FD_ = oral reference dose (mg kg^−1^ day^−1^), *T*_A_ = average life exposure time (365 days year^−1^ × lifetime, assuming 70 years), and SF = oral cancer slope factor (mg kg^−1^ day^−1^) ^−1^.

Concentrations used in the present study of risk calculations are on a wet weight basis. Oral reference dose (*R*_FD_) and oral cancer slope factor (SF) values were used from US EPA [45] for risk assessment.

## 3 Results and discussion

### 3.1 Concentration of pesticides in water and fish samples

In the present study eight pesticides viz chlordane, dimethoatte, malathion, atrazine, heptachlor, dichlorvos, azinphosmethyl and cypermethrin were detected. Table 2 depicts the results of sample analysis as mean value (μg/l) concentration of pesticides. The observed concentration of chlordane and heptachlor were 0.104 μg/l and 0.006 μg/l respectively. The concentration of other pesticides was dimethoate 0.082 μg/l, cypermethrin 0.076 μg/l, azinphosmethyl 0.065 μg/l, dichlorvos 0.059 μg/l, malathion 0.055 μg/l and atrazine 0.051 μg/l. The detected concentration of malathion in the present study was quite low from as reported earlier by (Sankaramakrishnan et al. [16] and Malik et al. [47]) from Kanpur and Lucknow sites of river Ganga. The newly introduced pesticides azinphosmethyl, dimethoate, atrazine, dichlorvos and cypermethrin were also detected in the present study as they are being continuously and abundantly used along the basin [8,48]. Rehana et al. [49] reported dimethoate in river Ganga from the same site, while Agnihotri et al. [50] reported heptachlor near Farrukhabad. Detection of malathion, heptachlor and chlordane shows their historical use and persistence in the ecosystem [51]. In accordance with the European Economic Commission (EEC Directive 80/778/EEC) [52] for drinking water the total pesticide level should not exceed 0.5 μg/l and the individual pesticide not more than 0.1 μg/l. The detected concentrations of the above mentioned pesticides were within the range of EEC limit except for chlordane which exceeds the limit. The detected concentration of the pesticides could be attributed to the agricultural runoff resulting from extensive agricultural activities along the basin.

**Table 2:** The concentration of pesticide residues in water (μg/l) and fish tissues (μg/g ww) from river Ganga, India.

Pesticide	LogK_ow_	Surface water	*Rita rita*	*Cyprinus carpio*
			Muscle tissues	
Chlordane	6.16	0.104 ± 0.33 (0.54−0.301)	0.101 ± 0.25 (0.67−0.278)	0.091 ± 0.52 (nd-0.187)
Dimethoate	0.78	0.082 ± 0.15 (0.051−0.191)	0.069 ± 0.64 (0.063−0.079)	0.079 ± 0.64 (0.071−0.095)
Malathion	2.36	0.055 ± 0.80 (nd-0.109)	0.081 ± 0.32 (0.075−0.097)	0.017 ± 0.71 (nd-0.111)
Atrazine	2.61	0.051 ± 0.19 (nd-0.104)	0.131 ± 0.19 (0.076−0.189)	0.087 ± 0.23 (0.055−0.104)
Heptachlor	6.10	0.006 ± 0.20 (nd-0.0024)	0.167 ± 0.20 (0.143−0.201)	0.142 ± 0.20 (0.123−0.209)
Dichlorvos	1.43	0.059 ± 0.17 (nd-0.132)	0.142 ± 0.11 (0.111−0.267)	0.163 ± 0.13 (0.109−0.267)
Azinphosmethyl	2.75	0.065 ± 0.80 (0.055−0.102)	0.045 ± 0.10 (nd-0.201)	0.138 ± 0.19 (0.117−0.197)
Cypermethrin	6.60	0.076 ± 0.93 (0.061−0.097)	0.052 ± 0.47 (nd-0.177)	0.182 ± 0.52 (0.154−0.235)

Data shown as mean ± standard deviation; maximum and minimum concentrations are in parenthesis.

Pesticide concentration detected in fish samples is given in Table 2. Mean concentrations (wet weight, ww) ranged from 0.167 μg g^−1^ for heptachlor to 0.045 μg g^−1^ for azinphosmethyl in bagrid *R rita*. Mean concentration in *C carpio* ranged from 0.182 μg g^−1^ for dichlorvos to 0.017 μg g^−1^ for malathion. The concentration of other pesticides were dichlorvos 0.142 μg/l, atrazine 0.131 μg/l, chlordane 0.101 μg/l, malathion 0.081 μg/l, cypermethrin 0.076 μg/l and dimethoate 0.069 μg/l in *R. rita*. In *C. carpio* the concentration of these pesticides were dichlorvos 0.163 μg/l, heptachlor 0.142 μg/l, azinphosmethyl 0.138 μg/l, atrazine 0.087 μg/l, chlordane 0.091 μg/l and dimethoate 0.079 μg/l. These results are in partial agreement with those obtained by [53], who found atrazine and chlorpyriphos residues in the muscle tissue of Tilapia fish collected from Rosetta Nile branch, Egypt. Dimethoate and malathion in the present study were reported lower than the earlier findings of Akhtar et al. [54]. Similarly, heptachlor and chlordane were reported lower than the value as reported earlier by Samanta [55] in fish tissue from the river Ganga at West Bengal site. Due to continuous exposure, contaminants accumulate and get concentrated in the muscle tissues compared to water. Various parameters influence the bioaccumulation of pesticides in fish, including water solubility, degree of ionization, stability, and size or shape of the chemical, and lipid content of the species [56]. The differences between pesticide concentrations in fishes from this study can be attributed to these factors and to differences in exposure. From the data presented in Table 2, it may be concluded that pesticide concentrations were lower in the omnivorous species (*C. carpio*) than in carnivorous species (*R. rita*).

### 3.2 Bioaccumulation factors

The bioaccumulation factor is the ratio of given chemical contaminant found inside the tissue of the fish to that found in the surrounding water. Pesticides being lipophilic, the detected concentration inside the tissue is normalized with lipid content. The present study shows, the BAF of malathion was found higher in both the selected fishes *R. rita* and *C. carpio*. Chlordane, dimethoate, atrazine, azinphosmethyl and cypermethrin in *R. rita* showed more accumulation than in *C. carpio*. Higher BAF might be accredited to poor water solubility and relative high log K _ow_ values (Table 2) [57,58]. From the studies conducted, it is known that bioaccumulation of chemical contaminant in the fish is due to the cumulative effect of many physiological and environmental conditions such as fish species, fish age, total lipid content including the environmental concentration of the contaminant [59,60]. BAF is always accessed by the hypothesis that the fish remains in the steady state in the surrounding environment, however it is not possible in the natural conditions. Further river Ganga is subjected to dynamic conditions because of large anthropogenic activities. Therefore, lipid content is not the only factor responsible for varied BAF difference but the total conditions of contexture of water.

### 3.3 Exposure assessment and risk characterization

Fish consumption is one of the most common sources of pesticide entry into human body [61]. Pesticide residues have a high potential negative effect on consumers [62,63]. Estimated daily intake (EDIs) was calculated to assess potential pesticide exposure to humans using mean values of each in the fish tissue as given in Table 2. Table 3 shows comparison between calculated EDIs and the acceptable daily intake (ADI) value issued by regulatory agency US EPA [45]. For the risk characterization all the chemicals were grouped into adversity groups based on chemical specific adversity and use of Rfd. From the assumption, the daily exposure to pesticides via consumption of these fish from the selected site of river Ganga, the potential risks in human are certain (Table 4). However, the target health quotient (THQs) values for both the fishes were less than 1.0, which indicate lower risk of eating fish from the study area. The calculated R (< 1 × 10^−4^) value associated with consumption of pesticide contaminated fish indicates negligible cancer risk US EPA [45]. However, the calculated R values of heptachlor were found to be higher than 1 × 10^−4^ for both the fishes indicating the heptachlor associated risk of cancer. Moreover, human behaviour including dietary habits varies greatly based on various criteria such as locality and socioeconomic status. Furthermore when considering specific single chemicals or assessment groups, such as pesticides in our case, we cann determine just a part of the overall risk and cannot provide an integrated assessment of the multiple risks triggered by exposure to different toxic stimuli [[64], [65], [66], [67]]. In this study grouping of the chemicals on adversity groups was based on common adversities instead of chemical structure or common mode of action approach that might lead to the underestimation of the real risk from multiple exposure to chemicals. It is certified by many studies that combined exposure from many sources, even at low levels can lead in time to unexpected toxic mixture effects [68,69]. Previous studies also suggested that various pesticides potentially pose health risk to the populations [70,71,72].

**Table 3:** Health hazard index for pesticide in fish *Rita rita* and *Cyprinus carpio*.

Name of pesticide	ADI (μg/kg/d) (US EPA)	EDI (μg/kg/d)	
		*Rita rita*	*Cyprinus carpio*
Chlordane	0.016	0.079	0.071
Dimethoate	0.01	0.054	0.062
Malathion	0.007	0.063	0.013
Atrazine	0.005	0.102	0.068
Heptachlor	0.002	0.131	0.111
Dichlorvos	0.002	0.111	0.128
Azinphosmethyl	0.001	0.035	0.108
Cypermethrin	0.02	0.040	0.143

**Table 4:** Non-carcinogenic (THQ) and carcinogenic risks (R) of pesticide residues.

Pesticide	Rfd (mg/kg/day)^−1^ (US EPA)	SF (mg/kg/day)^−1^ (US EPA)	THQ		R	
			*Rita rita*	*Cyprinus carpio*	*Rita rita*	*Cyprinus carpio*
Chlordane	0.00006	0.35	1.36E06	1.31E04	2.87E05	4.39E05
Dimethoate	0.0002		2.59E01	2.97E01		
Malathion	0.02	0.0038	3.05E03	6.40E04	2.31E07	4.86E08
Atrazine	0.035	0.22	2.81E03	1.87E03	2.17E05	1.44E05
Heptachlor	0.0005	4.5	2.51E01	4.49E06	5.66E04	4.81E04
Dichlorvos	0.0005	0.29	2.13E01	2.45E01	3.10E05	3.56E05
Azinphosmethyl	0.2		3.39E04	1.03E04		
Cypermethrin	0.01		3.91E03	1.37E02		

## 4 Conclusion

The present study provides a broad overview of pesticide status in water and two different edible fishes of the river Ganga. Surface water contains pesticide residue in quite detectable limit with mean concentration of chlordane 0.104 μg/l and heptachlor of 0.006 μg/l. Bagrid catfish *R. rita* tends to accumulate more residues than common carp *C. carpio*. Bioaccumulation factor was calculated for pesticides and found to be higher for malathion than other pesticides in both fishes. Lower than 1.0 target health quotient (THQ) implies lower non-carcinogenic risk via consumption of these fishes. Nevertheless, for heptachlor, exposure to carcinogenic risk are high. In summary, due to enormous use of pesticides in the agricultural field basin along the river, potential health risk associated with fish consumption cannot be ignored. The total residue limit should not exceed more than 0.1 μg/g [US EPA]. Further the fish is an important dietary source of proteins and possesses therapeutic values thus, effective measures need to be taken that help reduce total pesticide consumption along the basin and contamination of the fish.

## Author’s statement

Data will be available on request to the authors.

## Data availability statement

The datasets generated during and/or analysed during the current study are available from the corresponding author on reasonable request.

## Declaration of Competing Interest

The authors declare that they have no known competing financial interests or personal relationships that could have appeared to influence the work reported in this paper.
